# Femoral blood gas analysis, another tool to assess hemorrhage severity following trauma: an exploratory prospective study

**DOI:** 10.1186/s13049-023-01095-9

**Published:** 2023-06-20

**Authors:** Marie Werner, Benjamin Bergis, Pierre-Etienne Leblanc, Lucille Wildenberg, Jacques Duranteau, Bernard Vigué, Anatole Harrois

**Affiliations:** 1 Service d’Anesthésie Réanimation Chirurgicale, DMU 12 Anesthésie Réanimation Chirurgicale Médecine Péri-Opératoire et Douleur, Hôpital Bicêtre, AP-HP, Université Paris-Saclay, Équipe DYNAMIC, Inserm UMR_S999, Le Kremlin-Bicêtre, France; 2 Service d’Anesthésie Réanimation Chirurgicale, DMU 12 Anesthésie Réanimation Chirurgicale Médecine Péri-Opératoire et Douleur, Hôpital Bicêtre, AP-HP, Le Kremlin-Bicêtre, France

**Keywords:** Veno-arterial carbon dioxide tension difference, Venous oxygen saturation, Lactate, Tissue hypoxia, Severe trauma, Hemorrhagic shock

## Abstract

**Background:**

Veno-arterial carbon dioxide tension difference (ΔPCO_2_) and mixed venous oxygen saturation (SvO_2_) have been shown to be markers of the adequacy between cardiac output and metabolic needs in critical care patients. However, they have hardly been assessed in trauma patients. We hypothesized that femoral ΔPCO_2_ (ΔPCO_2 fem_) and SvO_2_ (SvO_2 fem_) could predict the need for red blood cell (RBC) transfusion following severe trauma.

**Methods:**

We conducted a prospective and observational study in a French level I trauma center. Patients admitted to the trauma room following severe trauma with an Injury Severity Score (ISS) > 15, who had arterial and venous femoral catheters inserted were included. ΔPCO_2 fem,_ SvO_2 fem_ and arterial blood lactate were measured over the first 24 h of admission. Their abilities to predict the transfusion of at least one pack of RBC (pRBC_H6_) or hemostatic procedure during the first six hours of admission were assessed using receiver operating characteristics curve.

**Results:**

59 trauma patients were included in the study. Median ISS was 26 (22–32). 28 patients (47%) received at least one pRBC_H6_ and 21 patients (35,6%) had a hemostatic procedure performed during the first six hours of admission. At admission, ΔPCO_2 fem_ was 9.1 ± 6.0 mmHg, SvO_2 fem_ 61.5 ± 21.6% and blood lactate was 2.7 ± 1.9 mmol/l. ΔPCO_2 fem_ was significantly higher (11.6 ± 7.1 mmHg vs. 6.8 ± 3.7 mmHg, *P* = 0.003) and SvO_2 fem_ was significantly lower (50 ± 23 mmHg vs. 71.8 ± 14.1 mmHg, *P* < 0.001) in patients who were transfused than in those who were not transfused. Best thresholds to predict pRBC_H6_ were 8.1 mmHg for ΔPCO_2 fem_ and 63% for SvO_2 fem_. Best thresholds to predict the need for a hemostatic procedure were 5.9 mmHg for ΔPCO_2 fem_ and 63% for SvO_2 fem_. Blood lactate was not predictive of pRBC_H6_ or the need for a hemostatic procedure.

**Conclusion:**

In severe trauma patients, ΔPCO_2 fem_ and SvO_2 fem_ at admission were predictive for the need of RBC transfusion and hemostatic procedures during the first six hours of management while admission lactate was not. ΔPCO_2 fem_ and SvO_2 fem_ appear thus to be more sensitive to blood loss than blood lactate in trauma patients, which might be of importance to early assess the adequation of tissue blood flow with metabolic needs.

**Supplementary Information:**

The online version contains supplementary material available at 10.1186/s13049-023-01095-9.

## Background

Severe trauma remains the leading cause of death before the age of 50 [1]. Approximately 40% of trauma deaths involve hemorrhagic shock and are possibly preventable through damage control resuscitation and early bleeding control [2, 3]. Hemorrhage-induced hypovolemia leads to a drop in cardiac output (CO) responsible for tissue hypoperfusion and impaired oxygen delivery to the organs. When the adaptive mechanisms to hypovolemia are overwhelmed and can no longer compensate for the decrease in oxygen delivery, alterations of cellular homeostasis may ultimately lead to refractory shock and multiorgan dysfunction [4]. It is therefore necessary to identify patients with severe blood loss as early as possible in order to quickly start appropriate resuscitation.

At the initial phase of severe trauma management, CO monitoring is not readily available. Several biomarkers have been proposed to assess the adequacy between oxygen delivery and demand in these conditions. Thus, admission lactate has been shown to be predictive of severe hemorrhage associated with massive transfusion [5]. However, during hemorrhage, blood lactate increases only beyond a critical threshold of oxygen delivery when cell anaerobic metabolism is triggered to maintain ATP production. Lactate production therefore only rises when blood loss gets significant [6]. Other metabolic parameters have been proposed to assess the adequacy between oxygen demand and supply in different critical conditions such as sepsis and major surgery. These parameters include the venous to arterial carbon dioxide gradient (ΔPCO_2_) [7, 8] and venous oxygen saturation (SvO_2_) [9, 10] but none of them has been studied in severe trauma patients. A drop in CO is responsible for a decrease in tissue blood flow leading to venous carbon dioxide accumulation and an increase in the fraction of extracted oxygen that cause an increase in ΔPCO_2_ [11] and a decrease in SvO_2_ respectively [12].

Femoral arterial and central venous catheters are commonly inserted during initial management of severe trauma patients in the trauma bay [13]. They make venous and arterial blood gas available to appraise the venous to arterial difference in CO_2_ (ΔPCO_2 fem_) as well as femoral venous oxygen saturation (SvO_2 fem_).

The aim of the present study was to assess the ability of ΔPCO_2 fem_, SvO_2 fem_ and arterial blood lactate to predict transfusion of red blood cells (RBC) over the first hours following severe trauma.

## Methods

### Study design

This observational, prospective and single-centre study was conducted in the surgical Intensive Care Unit of Bicêtre Hospital, an academic level-1 trauma center, from September 2015 to May 2020. This hospital provides 24-h availability of all essential trauma specialties, staff and equipment for trauma patient care. This study was approved by the ethics committee (“Comité de Protection des Personnes”) of the hospital (SC13-014 RCB: 2013-A01171-44) with waiver of participant consent. Patients or relatives were informed and we obtained confirmation of non-objection to data use.

### Study population

Patients over 18, admitted from the trauma scene, with an Injury Severity Score (ISS) greater than 15, and for whom initial management in the trauma room required the insertion of an arterial and a venous catheter at the femoral site, were included.

Pregnant women, patients with NYHA III or IV heart failure or chronic kidney disease (clearance < 30 ml/min) and patients with lower limb amputation or severe crush injury on the same side the femoral catheters were inserted were not included in the study.

### Clinical management

Following the emergency call, a physician-staffed mobile intensive care unit was sent to the trauma scene. After clinical assessment and medical care adjusted to trauma severity, patients were transported to the study center. Upon arrival in the trauma room, the insertion of femoral catheters was decided based on the patient’s history, clinical examination, and FAST ultrasound results, at the discretion of the clinician in charge as detailed elsewhere [13]. Briefly, the catheter lines were prepared in a sterile manner before patients’ arrival. Arterial (5 Fr, 11 cm) and venous femoral catheters (7 Fr, 20 cm) were simultaneously inserted by a trained resident under supervision of the trauma leader (consultant). The decision to transfuse was made by the physician in charge, based on his evaluation of the patient’s clinical situation using standard parameters (including clinical signs of shock, positive FAST ultrasound or obvious external hemorrhage and bed-side measurement of hemoglobin level) and blinded to the ΔPCO_2 fem_, SvO_2 fem_ results.

### Data collection

For each patient, demographics, past medical history, trauma characteristics, pre-hospital management data such as time to hospital admission, initial Glasgow Coma Scale (GCS) score, minimum systolic blood pressure, peripheral oxygen saturation, maximum heart rate, need for mechanical ventilation, volume of fluid resuscitation, catecholamines use were collected. We also reported the following scores: Injury Severity Score (ISS) [14], details of injuries for each organ (Abbreviated Injury Scale 2015, AIS) [15] and simplified acute physiology score (SAPS II) [16]. Severe organ injury was defined by an AIS for the correspondent organ over 2. On hospital admission, mean arterial blood pressure, heart rate, peripheral oxygen saturation, laboratory parameters (hemoglobin, prothrombin time, fibrinogen, myoglobin) were reported. Fluid administration, dose of catecholamines, transfusion therapy (type of product and amount) and hemostatic procedures were collected during the first 6 h of admission. The following outcome variables were also reported: length of mechanical ventilation, length of intensive care unit (ICU) stay and hospital mortality.

### Blood gas measurement

Venous and arterial blood gas were sampled from femoral catheters at insertion and regularly during the first 24 h, between 4 and 8 h from ICU admission (H6), 10 and 14 h from ICU admission (H12) 20 and 26 h from ICU admission (H24). pH, arterial (PaCO_2fem_) and femoral venous carbon dioxide partial pressure (PvCO_2fem_), arterial oxygen partial pressure (PaO_2_), venous oxygen saturation (SvO_2fem_), arterial oxygen saturation (SaO_2_)_,_ hemoglobin concentration and blood lactate were measured on both samples using a point-of-care blood gases analyzer (ABL 800, Radiometer). The femoral venous to arterial difference in carbon dioxide pressure (ΔPCO_2 fem_) was calculated as femoral PvCO_2_–PaCO_2_.

### Outcome

The primary outcome of interest was the transfusion of at least one pack of RBC during the first six hours of admission (pRBC_H6_). The secondary outcome was the need for an emergency hemostatic procedure. It was defined as performing angioembolization or hemostatic surgery within the first six hours of management from admission. Orthopedic surgery for fracture osteosynthesis was not considered as a hemostatic procedure.

There is no data regarding ΔPCO_2 fem_ and SvO_2fem_ in trauma patients. Since trauma patients present a wide variety of traumatic injuries, we considered that a sample of 60 patients would be representative for this exploratory study.

### Statistical analysis

Qualitative variables were expressed as counts (proportions). Quantitative variables were expressed as mean (SD) or median (25th–75th interquartile range) according to their distributions. Correlations between blood lactate, ΔPCO_2 fem_ and SvO_2 fem_ at admission were analyzed by Pearson correlation.

In the overall population, volumes of pRBC_H6_ transfused were reported according to quartile distribution of blood lactate, ΔPCO_2 fem_ and SvO_2 fem_. Then, patients were separated into two groups according to whether they had been transfused with pRBC_H6_ or not. Their characteristics were compared by a Student t-test or a Mann–Whitney test for quantitative variables (according to data distribution) and by a Chi-square test for qualitative variables. Evolution of lactate, ΔPCO_2 fem_ and SvO_2 fem_ during the 24 first hours of admission were compared over time and between the two groups with a mixed-effect model. Time, group (transfusion, no transfusion) and interaction (time x group) were set as fixed effect and patient as random effect.

To evaluate the ability of admission lactate, ΔPCO_2 fem_ and SvO_2 fem_ to predict the need for pRBC_H6_ transfusion or an emergency hemostatic procedure, receiver operating characteristic curves (ROCs) were built and their areas under the curve (AUC) were calculated. The best threshold for the prediction of pRBC_H6_ transfusion or to predict the need for an emergency hemostatic procedure was defined as the value maximizing the Youden index (Y = Sensitivity + Specificity-1). Sensitivity, specificity, positive and negative predictive values (PPV and NPV), were reported for each variable. Two-sided level of significance was fixed at *P* = 0.05. Data were analyzed using Prism (GraphPad Software, San Diego, California, USA).

## Results

### Population characteristics

Seventy patients were enrolled in the study. Eleven patients were secondarily excluded because their ISS was less than 15, leaving 59 patients in the final analysis (Additional file 1). Patients were 46 ± 20 years old and one third were women (17(29%)). Patients experienced predominantly blunt trauma (87%) with a median ISS of 26 (22–32). Median ICU length of stay was 10 (5–15.5) days and the overall hospital mortality was 26%. Two patients died in the first 24 h. The main characteristics of the population are presented in Table 1.

**Table 1:** General characteristics in the overall population and in both transfused and non-transfused patients

	All	Non-transfused	Transfused^ε^	*P*^#^
	n = 59	n = 31	n = 28	
Characteristics				
Age-years	46 ± 20	47 ± 21	45 ± 19	0.7
Women-n (%)	17 (29)	10 (32)	7 (25)	0.6
Type of trauma-n (%)				0.4
Road accident	35 (59.3)	18 (58)	17 (61)	
Fall from height	19 (32.2)	11 (35.5)	8 (28.5)	
Penetrating trauma	2 (3.4)	0 (0)	2 (7)	
Other	3 (5.1)	2 (6.5)	1 (3.5)	
ISS	26 (22–32)	27 (25–35)	25 (21–30)	0.1
AIS^ϕ^ Head or neck-n (%)	28 (47)	20 (64.5)	8 (28.6)	0.006*
AIS Chest-n (%)	32 (54)	20 (64.5)	12 (42.9)	0.1
AIS Abdomen-n (%)	15 (25)	3 (9.6)	12 (42.9)	0.003*
AIS Pelvis and extremities-n (%)	24 (41)	9 (29)	15 (53.6)	0.02*
AIS External-n (%)	2 (1)	0 (0)	1 (4)	0.3
SAPS II	43 (20)	45 (20)	40 (20)	0.4
On scene parameters				
Time from trauma to hospital admission-min	98 (67–120)	105 (74–122)	95 (57–113)	0.3
GCS	14 (8–15)	13 (6–15)	15 (13–15)	0.03*
Minimum SBP-mmHg	106 ± 33	104 ± 39	107 ± 24	0.7
Maximum HR-bpm	106 ± 25	102 ± 27	108 ± 23	0.4
Minimum SpO_2_-%	98 (94–98)	97 (90–98)	99 (95–100)	0.02*
Intubation-n(%)	32 (54.2)	20 (64.5)	12 (42.9)	0.09
Fluid administration-ml	800 (500–1250)	1000 (500–1250)	750 (500–1038)	0.2
Catecholamines-mg/h	0 (0–0.4)	0 (0–0.5)	0 (0–0.25)	0.9
Parameters on admission to resuscitation room				
MBP H0-mmHg	80 ± 19	85 ± 16	73 ± 19	0.02*
HR H0-bpm	98 ± 26	93 ± 23	102.6 ± 27	0.2
Ht-%	0.34 ± 0.06	0.37 ± 0.04	0.31 ± 0.6	< 0.001*
Hb-g/dl	11.5 ± 2.1	12.6 ± 1.5	10.3 ± 2	< 0.001*
PT-%	70 ± 17	75 ± 14	64 ± 18	0.01*
Fibrinogen-g/l	2 ± 1	2.4 ± 0.8	2.0 ± 1.0	0.1
Myoglobin-IU	945 (386–2597)	910 (425–1762)	1151 (350–2722)	0.8
Outcome during hospital stay				
Crystalloids H6-ml	1750 (875–3500)	1000 (500–1870)	3125 (1575–4137)	< 0.001*
Colloids H6-ml	0 (0–500)	0 (0–0)	0 (0–500)	0.02*
RBC H6-ml	0 (0–892)	0	900 (505–1243)	< 0.001*
Catecholamines H6-mg/hour	1.0 (0.3–2.5)	0.6 (0.25–2.5)	1 (0–2.6)	0.8
RBC H6-H12-ml	0 (0–0)	0 (0–0)	0 (0–70)	0.004*
Hemostatic procedure-n	21 (35.6)	2 (6.5)	19 (67.8)	< 0.001*
Mortality-n	15 (25.9)	11 (35.5)	4 (14.8)	0.06

AIS^ϕ^ is the number of patients with an AIS > 2. Hemostatic procedure was defined as performing angioembolization or hemostatic surgery within the first six hours of management from admission

Data are reported as mean ± SD or median [Q1–Q3]

*GCS* glasgow coma scale, *Hb* hemoglobin, *HR* heart rate, *Ht* hematocrit, *H6* Over the first 6 h after admission, *H6-H12* between H6 and H12 after admission, *ICU* intensive care unit, *ISS* injury severity score, *MBP* mean blood pressure, *PT* prothrombin time, *RBC* red blood cells, *SAPS* simplified acute physiology score, *SD* standard deviation, *SBP* systolic blood pressure, *SpO*_2_ pulse oximeter oxygen saturation

^ε^Patient transfused with red blood cells over the first six hours of admission

^#^Non-transfused patients were compared with transfused patients

^*^Two-sided level of significance was fixed at 5%

### Incidence of transfusion

Over the first six hours of admission, 28 patients (47%) received at least one pack of RBC. The characteristics of transfused and non-transfused patients are shown in Table 1. Transfused and non-transfused patients were similar with regard to age, gender, SAPSII, type of trauma and on-scene hemodynamic parameters. Transfused patients presented more severe abdominal (43% vs. 10%, *P* = 0.006) and pelvic trauma (54% vs. 29%, *P* = 0.020), but they had significantly less traumatic head injuries (28.6% vs. 64.5%, *P* = 0.006) than non-transfused patients.

Mean blood pressure at hospital admission was significantly lower in the transfused group (*P* = 0.020). The given volume of crystalloids (*P* < 0.001) and colloids (*P* = 0.020) over the first six hours of management were significantly greater in patients who got transfused. Nineteen patients (67.8%) required an emergency hemostatic procedure in the transfused group and only 2 (6.5%) in the non-transfused group (*P* < 0.001). Mortality was not different between the two groups (*P* = 0.060) (Table 1).

### Blood gas analysis

**Fig. 1:**
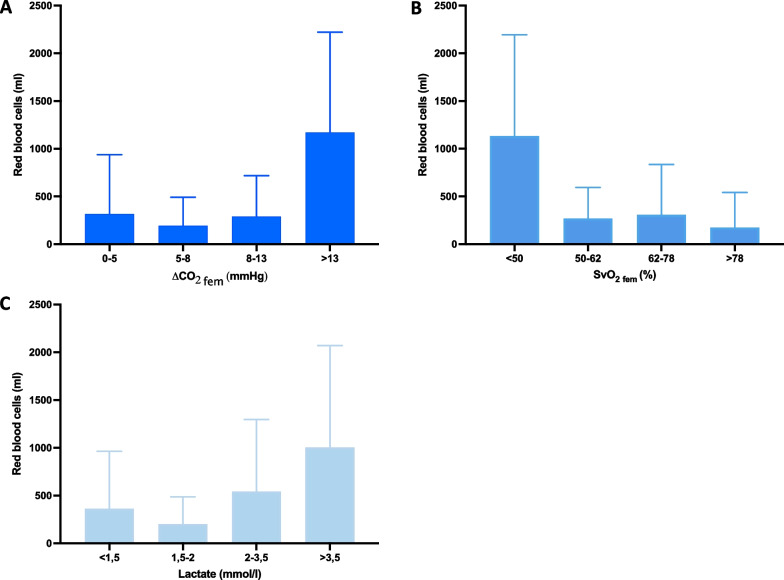
Volumes of red blood cells transfused during the first six hours of admission. Volumes are shown according to quartile distribution of **A** ΔPCO_2 fem_. **B** SvO_2 fem_ and **C** lactate. All data are reported as mean ± SD. ΔPCO_2 fem_ femoral venous-arterial difference in carbon dioxide pressure. SvO_2 fem_ femoral venous oxygen saturation

In the whole cohort, blood lactate was 2.7 ± 1.9 mmol/l, ΔPCO_2 fem_ was 9.1 ± 6.0 mmHg and SvO_2 fem_ was 61.5 ± 21.6% on admission. As shown in Fig. 1, volume of pRBC_H6_ was associated with a greater ΔPCO_2 fem_ or blood lactate level and a lower SvO_2 fem_. Volume of pRBC_H6_ transfused was larger when ΔPCO_2 fem_ was greater than 13 mmHg, SvO_2 fem_ was less than 50% or blood lactate was greater than 3.5 mmol/l (Fig. 1).

At admission, in the overall population ΔPCO_2 fem_ was at its highest level and then decreased to a mean minimal value at H24 of 5.4 ± 3.5 mmHg. SvO_2 fem_ was the lowest at admission and then plateaued from H6 with a mean value of 68.5 ± 16.8%. Maximum lactate was reached at H6 (2.8 ± 2.1 mmol/) and then decreased with a mean value of 2.0 ± 1.9 mmol/l at H24 (Additional file 1). At admission, ΔPCO_2 fem_ (r = 0.48; *P* < 0.001) and SvO_2 fem_ (r = 0.40; *P* < 0.004) were correlated with lactate and they were correlated together (r = 0.63; *P* < 0.001).

At admission, ΔPCO_2 fem_ (11.6 ± 7.1 mmHg vs. 6.8 ± 3.7 mmHg; *P* = 0.003), SvO_2 fem_ (50.0 ± 23.0% vs. 71.8 ± 14.1%; *P* < 0.001) and lactate (3.3 ± 2.4 mmol/l vs. 2.2 ± 1.3 mmol/l; *P* = 0.04) were significantly different in transfused compared to non-transfused patients (Table 2).

**Table 2:** Hemodynamic variables in the overall population and in transfused and non-transfused patients

	All	Not transfused	Transfused	*P*
	n = 59	n = 31	n = 28	
Lactate—mmol/L	2.7 ± 1.9	2.5 ± 1.9	3.1 ± 2.1	0.036
ΔPCO_2 fem_—mmHg	9.1 ± 6.0	6.8 ± 3.7	11.6 ± 7.1	0.003
SvO_2 fem_—%	61.5 ± 21.6	71.8 ± 14.1	50.0 ± 23.0	< 0.001

ΔPCO_2 fem_ femoral venous-arterial difference in carbon dioxide pressure. SvO_2 fem_ femoral venous oxygen saturation. Data are reported as mean ± SD

**Fig. 2:**
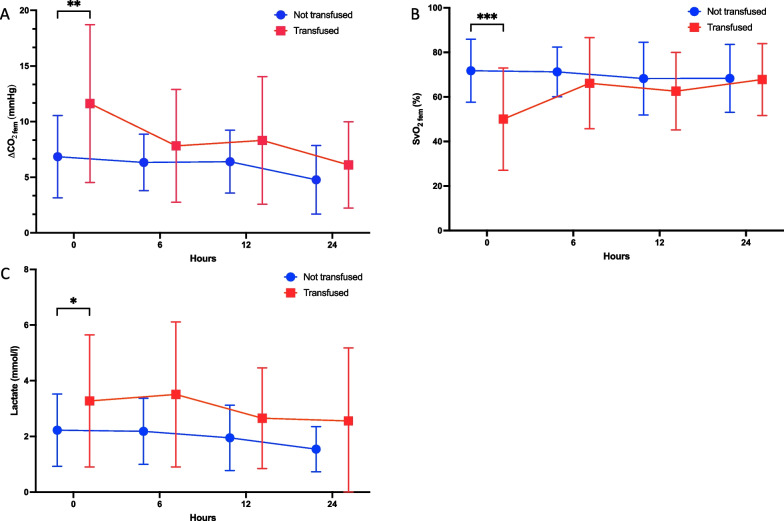
Evolution during the first 24 h of hemodynamic variables in transfused and non-transfused patients. **A** ΔPCO_2 fem_. **B** SvO_2 fem_ and **C** lactate. All data are reported as mean ± SD. *P < 0.05; **P < 0.005; ***P < 0.001. Parameters were measured at hospital admission and over the first 24 h. ΔPCO_2 fem_ femoral venous-arterial difference in carbon dioxide pressure. SvO_2 fem_ femoral venous oxygen saturation

Over the first 24 h, ΔPCO_2 fem_ decreased significantly and was statistically different between transfused and non-transfused groups without interaction between time and groups (time effect, *P* = 0.002; transfusion effect, *P* = 0.008; time x transfusion effect, *P* = 0.1). SvO_2 fem_ remained stable in the non-transfused group, over the first 24 h, while it significantly increased over time in the transfused group (time effect, *P* = 0.04; transfusion effect, *P* = 0.02; time x transfusion effect, *P* < 0.001). In the transfused group, blood lactate peaked at H6 and then decreased over the first 24 h while it decreased, from admission to H24, in the non-transfused group. Blood lactate was different between non-transfused and transfused patients without interaction between time and groups (time effect, *P* = 0.01; transfusion effect, *P* = 0.007; time x transfusion effect, *P* = 0.7) (Fig. 2 and Additional files 2 and 3).

### Transfusion prediction

**Fig. 3:**
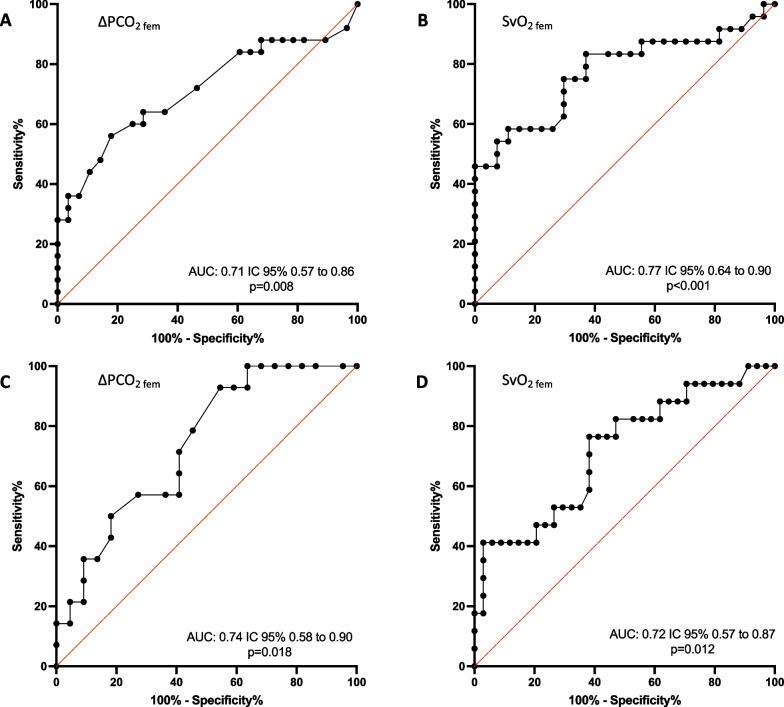
ROC curves for prediction of RBC transfusion and hemostatic procedure by ΔPCO_2fem_ and SvO_2fem_. **A** ROC curve for prediction of pRBC_H6_ by ΔPCO_2 fem_ at admission. **B** ROC curve for prediction of pRBC_H6_ by SvO_2 fem_ at admission. **C** ROC curve for prediction of hemostatic procedure during the first 6 h of admission by ΔPCO_2 fem_ at admission. **D** ROC curve for prediction of hemostatic procedure during the first 6 h of admission by SvO_2 fem_ at admission. AUC area under the curve. ΔPCO_2 fem_ femoral venous-arterial difference in carbon dioxide pressure. pRBC_H6_ transfusion of at least 1 pack of red blood cell during the first 6 h of admission. ROC Receiver operating characteristics. SvO_2 fem_ femoral venous oxygen saturation

The abilities of admission ΔPCO_2 fem_, SvO_2 fem_ and blood lactate to predict the need for pRBC_H6_ transfusion and the need for a hemostatic procedure during the first six hours of admission are presented in Fig. 3 and Additional file 4.

Admission ΔPCO_2 fem_ significantly predicted pRBC_H6_ transfusion with an AUC of 0.71 (CI95% 0.57–0.86; *P* = 0.008) and SvO_2 fem_ significantly predicted pRBC_H6_ transfusion with an AUC of 0.77 (CI95% 0.64–0.91; *P* < 0.001). At admission, the optimal thresholds of ΔPCO_2 fem_ to predict the need for pRBC_H6_ transfusion was 8.1 mmHg and the optimal threshold of SvO_2 fem_ to predict the need for pRBC_H6_ transfusion was 63%. The predictive performances of ΔPCO_2 fem_ and SvO_2 fem_ are given in Table 3.

**Table 3:** Predictive performances of hemodynamic variables for transfusion of red blood cells during the first six hours of admission

	AUC (95% CI)	*P*	Cutt-off value	Sensitivity (%)	Specificity (%)	PPV (%)	NPV (%)
Lactate	0.64 (0.5–0.79)	0.07	1.9 mmol/l	78.6	58.1	62.4	75.3
ΔPCO_2 fem_	0.71 (0.57–0.96)	0.008	8.1 mmHg	64	71.4	66.2	68.4
SvO_2 fem_	0.77 (0.64–0.9)	< 0.001	63%	75	70.4	69.7	75.7

ΔPCO_2 fem_ femoral venous-arterial difference in carbon dioxide pressure. SvO_2 fem_ femoral venous oxygen saturation

*NPV* negative predictive value. *PPV* positive predictive value of parameters

Admission ΔPCO_2 fem_ and SvO_2 fem_ significantly predicted the need for an emergency hemostatic procedure with respective AUCs of 0.74 (CI95% 0.58–0.90, *P* = 0.02) and 0.72 (CI95% 0.57–0.87, *P* = 0.01). A threshold of 5.9 mmHg for ΔPCO_2 fem_ and 63% for SvO_2 fem_ at admission were the optimal values to predict the need for an emergency hemostatic procedure.

Admission blood lactate did not significantly predict the need for pRBC_H6_ transfusion (AUC = 0.64 CI95% 0.49–0.79; *P* = 0.07) or the need for a hemostatic procedure (AUC = 0.60 CI95% 0.42–0.78; *P* = 0.3) in the first six hours of admission.

## Discussion

We performed this prospective and observational study to assess the ability of ΔPCO_2 fem_, SvO_2 fem_ and arterial blood lactate to predict transfusion of red blood cells over the first six hours (pRBC_H6_) following severe trauma. We found that the difference in femoral venous to arterial PCO_2_ (ΔPCO_2 fem_) and venous oxygen saturation (SvO_2 fem_) at admission were predictive of RBC transfusion within the first six hours of admission, with respective optimal thresholds of 8.1 mmHg and 63%. Second, we also found that ΔPCO_2 fem_ and SvO_2 fem_ were predictive of an emergency hemostatic procedure. Third, though admission blood lactate correlated with the volume of transfused RBC over the first 6 h, it was neither predictive of pRBC_H6_ transfusion nor predictive of an emergency hemostatic procedure. Fourth, ΔPCO_2 fem_ and SvO_2 fem_ normalized earlier than lactate after transfusion.

### Relationship with previous studies

Venous to arterial PCO_2_ difference and venous O_2_ saturation have hardly been studied in the setting of severe trauma. We found that admission ΔPCO_2 fem_ and SvO_2 fem_ were predictive of pRBC_H6_ transfusion. These results are consistent with studies carried out on animal models of hemorrhagic shock [17, 18] where incremental blood loss led to a progressive increase in ΔPCO_2_ and decrease in SvO_2_. According to Fick equation, generated CO_2_ (VCO_2_) equals the product of CO by the difference between mixed venous and arterial CO_2_ contents (Cv-aCO_2_) and is constant in aerobic and anaerobic conditions. ΔPCO_2_ has been shown to be linearly related to Cv-aCO_2_. Thus, increase in ΔPCO_2_ during hemorrhage results from a drop in CO leading to CO_2_ stagnation at tissue level with an increase in venous CO_2_. During hemorrhage, an increase in ΔPCO_2_ thus reflects the inadequacy between CO and metabolic activity. Hemorrhage also results in a decrease in oxygen transport to tissues responsible for an increase in oxygen extraction ratio and therefore a decrease in SvO_2_. Thus, increased ΔPCO_2 fem_ and decreased SvO_2 fem_ likely reflect the decrease in tissue blood flow to the lower limbs related to the magnitude of blood loss in trauma patients.

We measured femoral ΔPCO_2_ and SvO_2_ (ΔPCO_2 fem_ and SvO_2 fem_) while most clinical and experimental studies reported about central ΔPCO_2_ and ScvO_2_. Normal central ΔPCO_2_ ranges from 2 to 5 mmHg [19]. In a study conducted in 14 healthy volunteers, ΔPCO_2 fem_ ranged from 2 to 4 mmHg while volunteers were passively cycling [20]. Several studies reported conflicting differences between ScvO_2_ and SvO_2 fem_ with bias of 2.7 ± 7.9% in patients undergoing elective right heart catheterization [21] to bias of 2 to 8% in critical care patients [22, 23]. However, though SvO_2 fem_ is not a reliable surrogate of ScvO_2_, we found it to be more predictive of pRBC_H6_ transfusion than admission ΔPCO_2 fem_ or blood lactate.

Although there is a significant correlation between blood lactate, ΔPCO_2 fem_, SvO_2 fem_, these measurements are not interchangeable. Unlike what Régnier et al. reported, admission lactate was not found as a predictive marker of pRBC_H6_ transfusion [5]. However, we used a lower threshold (one pRBC_H6_ as compared to massive transfusion defined by 6 pack of RBC in 24 h). ΔPCO_2 fem_ and SvO_2 fem_ appeared thus to be more sensitive than blood lactate to predict hemorrhage requiring RBC transfusion. A hypothesis is that these two parameters are a direct reflection of tissue blood flow to the lower limbs while aerobic metabolism is still present [24], whereas blood lactate increases at a later stage of hemorrhage, when patient blood loss is more pronounced with concomitant triggering of anaerobic metabolism [6, 25]. Consistently with what has been shown in animals [6], following an active hemorrhage, it would seem that the drop in CO, responsible for peripheral vasoconstriction and a drop in blood flow to the lower limbs can be more rapidly detected by an elevation of ΔPCO_2 fem_ and a decrease of SvO_2 fem_ than using blood lactate level. Blood flow is indeed early reduced in musculo-cutaneous tissue (i.e. lower limbs) to preserve flow to the noble organs (i.e. heart, brain) [26], which may explain the early increase in ΔPCO_2 fem_ and decrease in SvO_2fem_.

It is interesting to note that patients did not present with the same injuries in the two groups. The transfused group presented mainly abdominal and pelvic injuries, more often responsible for hemorrhage, while the non-transfused group presented mainly intracranial injuries. Although mean ΔPCO_2 fem_ was higher in the transfused group compared to the non-transfused group (11.6 vs. 6.8 mmHg), some patients had a high ΔPCO_2 fem_ in the non-transfused group. These patients presented a severe head injury with an AIS head greater than 3, or had bowel perforation without hemorrhage and others had severe thoracic injuries with a hemo- or pneumothorax. In patients with no clinical or CT evidence of hemorrhage, the presence of a high ΔPCO_2 fem_ should therefore lead to the search for another cause of cardiovascular dysfunction. Severely injured trauma patients can indeed present causes of shock other than hemorrhage like tamponade, peritonitis associated with bowel perforation, spinal cord injury but also neurogenic cardiovascular dysfunction reported in several studies in patients with severe head injury [27].

Consistent with what has been observed in animals, ΔPCO_2 fem_ and SvO_2 fem_ corrected more rapidly than lactate, which increased until H6 in the transfused group [25]. ΔPCO_2 fem_ and SvO_2 fem_ are likely corrected in parallel with the improvement of tissue blood flow following transfusion, whereas hyperlactatemia is an unreliable marker of hypoxia and hypoperfusion. Indeed, lactate production is also a consequence of cellular dysoxia secondary to injury-induced inflammation and microcirculation alterations, frequently observed after severe trauma, despite tissue perfusion improvement [28]. Unrelated to tissue dysoxia, hyperlactatemia is also frequently observed in shock state, following aerobic glycolysis activation through catecholamine-dependent ß2-receptor stimulation [29].

### Implication of study findings

Our findings imply that ΔPCO_2 fem_ and SvO_2 fem_ are more sensitive than blood lactate to predict the need for early blood transfusion or hemostatic procedure, on admission of severe trauma patients. Seven patients (25%) who were transfused with RBC during the first six hours of management had indeed a high ΔPCO_2 fem_ and/or a low SvO_2 fem_ but a normal blood lactate. Moreover, the evolution of ΔPCO_2 fem_ and SvO_2 fem_ over time may help to assess transfusion effectiveness to restore adequate tissue perfusion. Cardiac output monitoring using standard tools such as cardiac ultrasound or thermodilution are not available in the trauma bay during the initial phase, and macro-hemodynamic parameters such as heart rate and mean blood pressure are poorly correlated with CO during hemorrhagic shock states. Thus, in this study, we showed that ΔPCO_2 fem_ and SvO_2 fem_ could be easily used to assess the adequation of tissue blood flow (i.e. to the lower limbs) with metabolic needs following severe trauma.

### Strengths and limitations

This study has several strengths. To our knowledge, this is the first study evaluating ΔPCO_2_ and SvO_2 fem_ in severe trauma patients and their capacities to predict early transfusion. Moreover, we provided new data on the level of ΔPCO_2 fem_ and SvO_2 fem_ values in such patients. These results allowed us to propose alternative markers to assess the adequacy between tissue blood flow and metabolic needs in trauma patients.

This study also has several limitations. First, it is a single-centre study with a small sample-size, in patients who required the insertion of an arterial and a venous catheter at the femoral site which prevents generalization of these results. Indeed, only patients which ICU clinician judged, severe at hospital admission [13], were included which make this study applicable only to these patients. Second, we studied ΔPCO_2_ and SvO_2_ at the femoral site which are not good surrogates of central ΔPCO_2_ and ScvO_2_, considered as standards. However, we were able to report cut-off values for these markers at femoral site for transfusion prediction. Third we do not know the normal values for ΔPCO_2 fem_ and SvO_2 fem_ at baseline but we highlighted significant differences in ΔPCO_2 fem_ and SvO_2 fem_ according to the need for transfusion, reflecting the hemorrhage-induced drop in tissue blood flow and allowing to assess resuscitation efficacy over time. Nevertheless, additional studies will be necessary to determine the range of normal value of ΔPCO_2 fem_ and SvO_2 fem_ at the femoral level. Finally, since SvO_2_ is dependent on hemoglobin, its predictive value for red blood cell transfusion may have been overestimated by the effect of anemia in patients with severe hemorrage [30]. Moreover, due to the Haldane effect, it is possible that in the most severe patients with low hemoglobin level and significant metabolic acidosis, the ΔPCO_2 fem_ may have been overestimated compared to veno-arterial CO_2_ content (Cv-aCO_2_). Indeed, for the same level of Cv-aCO_2_, ΔPCO_2_ increases in case of metabolic acidosis and anemia [31] making the relationship between the two values no longer linear. This was however observed at the late phase of hemorrhage, when bloodloss was major [17] and, in a way, reflect the seriousness of hemorrhage which is consistent with what we expected to predict.

## Conclusion

In severe trauma patients, ΔPCO_2_ and SvO_2_ measured at the femoral level at admission were predictive for the need of RBC transfusion and hemostatic procedures during the first six hours of management while admission lactate was not. In this study, ΔPCO_2 fem_ and SvO_2 fem_ appear thus to be more sensitive to blood loss than lactate in patients requiring femoral venous and arterial catheters insertion, which might be of importance to assess the adequation of tissue blood flow with metabolic needs following severe trauma.

## Supplementary Information

**Additional file 1**. Flow-chart of study participants.

**Additional file 2.** Evolution of ΔPCO_2 fem_, SvO_2 fem_ and lactate during the first 24 hours in the overall population. All data are reported as mean ± SD. Parameters were measured at hospital admission and over the first 24 hours. ΔPCO_2 fem_ femoral venous-arterial difference in carbon dioxide pressure. SvO_2 fem_ femoral venous oxygen saturation.

**Additional file 3**. Evolution of blood gas parameters during the 24 first hours of admission. Data are shown in the overall population in non-transfused and transfused patients. All data are reported as mean ± SD. Art pH arterial pH. H0 measurement at admission. H_6_ measurement 6 hours after admission. H12 measurement 12 hours after admission. H_2_4 measurement 24 hours after admission. ΔPCO_2_ fem femoral venous-arterial difference in carbon dioxide pressure. SvO_2_ fem femoral venous oxygen saturation.

**Additional file 4**. ROC curves for prediction of red blood cell transfusion and hemostatic procedure by lactate. **a** ROC curve for prediction of pRBCH6 by lactate at admission. **b** ROC curve for prediction of hemostatic procedure during the first 6 hours of admission by lactate at admission. AUC area under the curve. pRBCH6 transfusion of at least 1 pack of red blood cell during the first 6 hours of admission. ROC Receiver operating characteristics.

## Data Availability

After publication, the data will be made available upon reasonable request from the corresponding author.

## Abbreviations

ΔPCO_2_: Veno-arterial carbon dioxide tension difference
ΔPCO_2 fem_: Femoral veno-arterial carbon dioxide tension difference
AIS: Abbreviated Injury Scale 2015
CO: Cardiac output
GCS: Glasgow Coma Scale
Hb: Hemoglobin
HR: Heart rate
Ht: Hematocrit
ICU: Intensive care unit
ISS: Injury severity score
MBP: Mean blood pressure
PaCO_2_: Arterial carbon dioxide partial pressure
PaO_2_: Arterial oxygen partial pressure (PaO_2_)
pRBC_H6_: Pack of red blood cell transfused over the first 6 h of admission
PvCO_2_: Venous carbon dioxide partial pressure
PT: Prothrombin time
RBC: Red blood cells
ROC: Receiver operating characteristics
SAPS: Simplified Acute Physiology Score
SD: Standard deviation
SBP: Systolic blood pressure
SaO_2_: Arterial oxygen saturation
SpO_2_: Pulse oximeter oxygen saturation
SvO_2_: Venous oxygen saturation
SvO_2 fem_: Femoral venous oxygen saturation
