# Supplementary figures and images for: Femoral blood gas analysis, another tool to assess hemorrhage severity following trauma: an exploratory prospective study
Source: Scand J Trauma Resusc Emerg Med. 2023 Jun 20;31:31. doi: 10.1186/s13049-023-01095-9 (PMC10280927; doi:10.1186/s13049-023-01095-9)

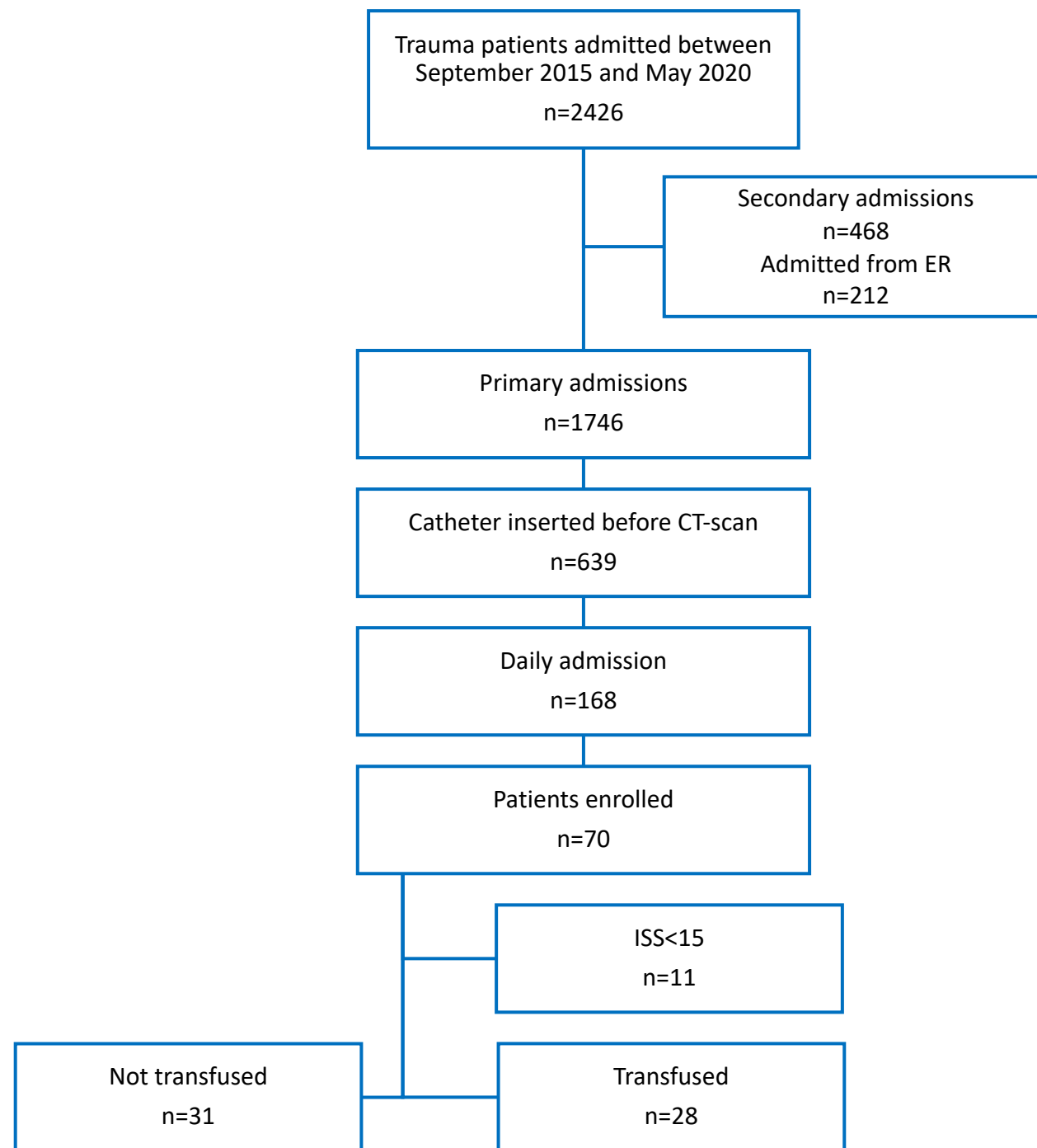

Supplement: Supplementary file 1 — Additional file 1. Flow-chart of study participants. [file 13049_2023_1095_MOESM1_ESM.pdf]

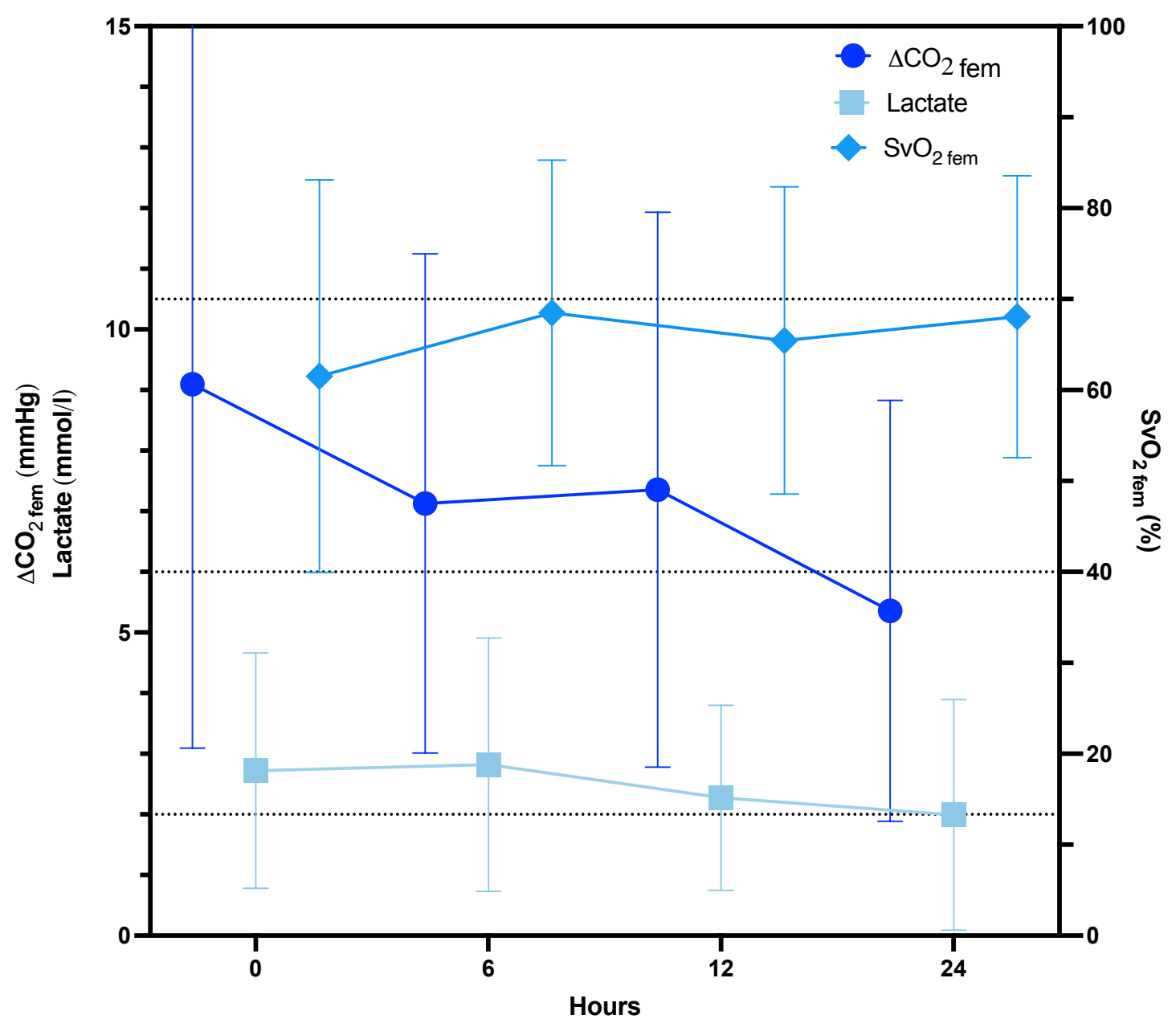

Supplement: Supplementary file 2 — Additional file 2. Evolution of ΔPCO2 fem, SvO2 fem and lactate during the first 24 hours in the overall population. All data are reported as mean ± SD. Parameters were measured at hospital admission and over the first 24 hours. ΔPCO2 fem femoral venous-arterial difference in carbon dioxide pressure. SvO2 fem femoral venous oxygen saturation. [file 13049_2023_1095_MOESM2_ESM.pdf]

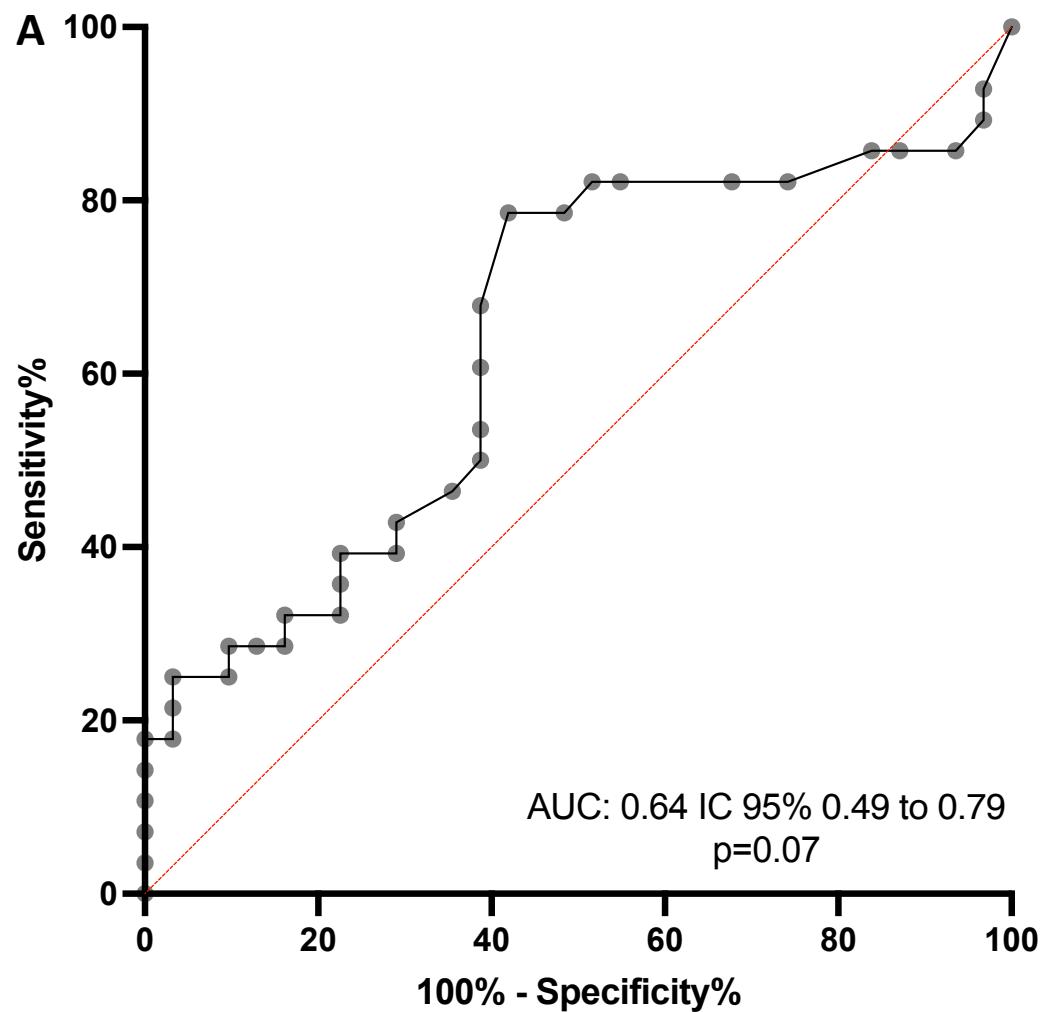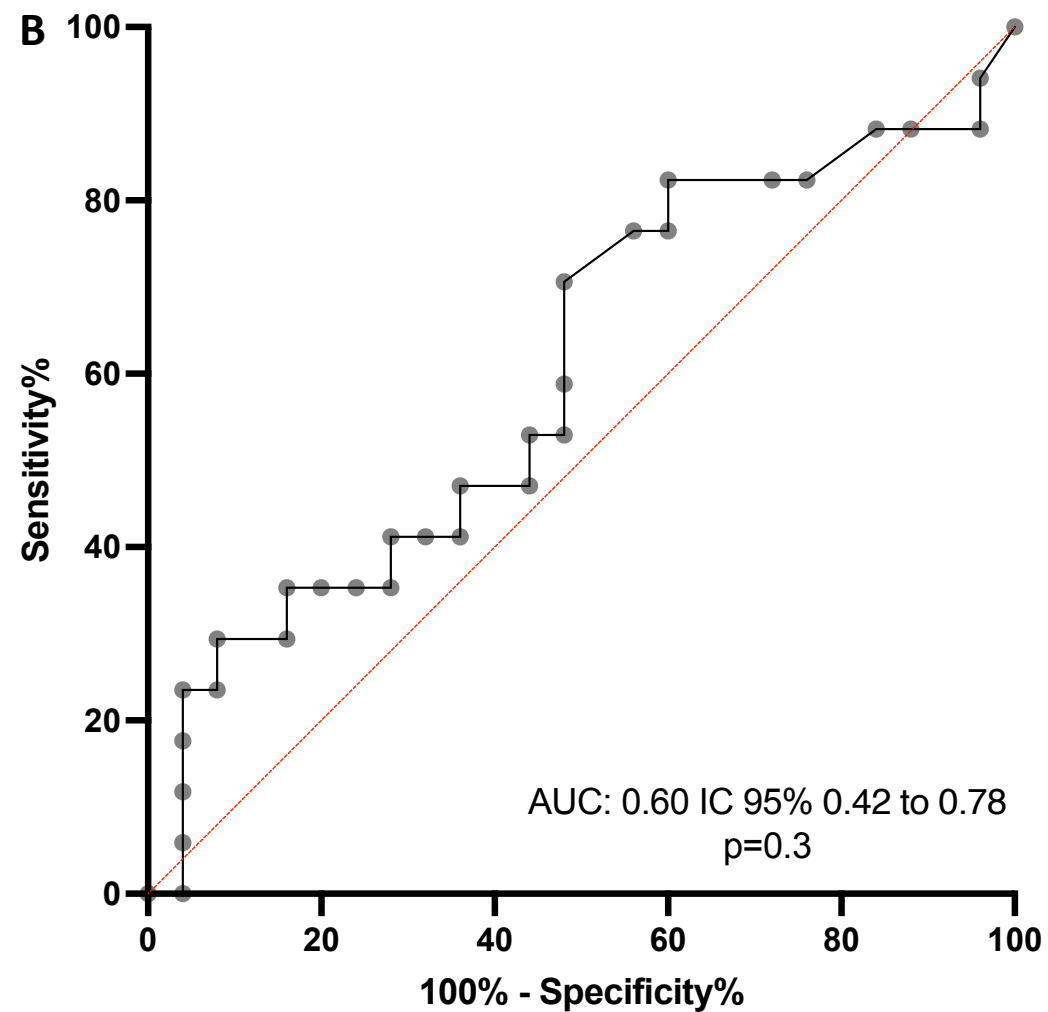

Supplement: Supplementary file 4 — Additional file 4. ROC curves for prediction of red blood cell transfusion and hemostatic procedure by lactate. a ROC curve for prediction of pRBCH6 by lactate at admission. b ROC curve for prediction of hemostatic procedure during the first 6 hours of admission by lactate at admission. AUC area under the curve. pRBCH6 transfusion of at least 1 pack of red blood cell during the first 6 hours of admission. ROC Receiver operating characteristics. [file 13049_2023_1095_MOESM4_ESM.pdf]
